# Can nitric oxide-responsive polymers enable precision therapeutics for ageing?

**DOI:** 10.17179/excli2026-9640

**Published:** 2026-08-20

**Authors:** Parteek Prasher, Mousmee Sharma

**Affiliations:** 1 Department of Chemistry, University of Petroleum & Energy Studies (UPES), Dehradun, 248007, India; 2 Department of Chemistry, Uttaranchal University, Dehradun, 248007, India

**Keywords:** nitric oxide, gasotransmitter, ageing, precision therapeutics, drug delivery

## Abstract

Disruptions in nitric oxide homeostasis, characterised by endothelial dysfunction, neurodegeneration, metabolic decline, and chronic inflammation represent a hallmark of the ageing process. These pathological features led to the development of NO-responsive polymeric systems which exploit the endogenous gasotransmitter fluctuations for offering therapeutic and diagnostic applications. This commentary highlights the advances in NO-responsive polymers, which undergo NO-triggered self-assembly or disassembly, fluorescence activation for intracellular NO imaging, NO scavenging, and site-specific drug release. Although critical challenges still persist, including tissue-specific variability in NO levels and validation in clinically relevant ageing models, the contemporary advances in polymer engineering and design aim to translate these intelligent biomaterials towards ageing-targeted precision therapeutics.

## ⁯

A decline in the cellular functioning and homeostasis is often marked by mitochondrial dysfunction, DNA damage, chronic inflammation (inflammaging), stem-cell senescence, and impaired protein homeostasis which gradually results in tissue degeneration, impairment of the organ function, and increased vulnerability to various diseases. Stimuli-responsive polymers represent a promising class of 'smart' biomaterials which are capable of responding to subtle changes in the cellular microenvironment. Such materials undergo a site-specific activation which enhances their therapeutic precision, thereby making them particularly attractive for addressing the complex biological challenges associated with ageing.

The contemporary stimuli-responsive 'smart' polymers include pH-responsive systems that undergo physicochemical transformations in the gastrointestinal tract, intracellular organelles, and tumour microenvironments; thermo-responsive polymers that exhibit LCST/UCST phase transitions in response to temperature fluctuations; and enzyme-responsive systems that undergo selective degradation upon interaction with disease-associated or inducible enzymes. Similarly, redox-responsive polymeric materials leverage on elevated glutathione and ROS in cancer and inflammation. Apart from this, other biological triggers such as glucose levels, ATP concentration, ionic strength, and hypoxia have also been widely investigated for targeted release of the payload molecule. Eventually, the external stimuli-triggered payload release has been achieved by light-responsive polymers, magnetic field-responsive polymeric nanocomposite materials, electrically responsive conductive polymers, ultrasound-sensitive polymeric carriers, and thermally/ mechanically sensitive polymeric materials (Shymborska et al., 2024[8]).

Among these materials, the gasotransmitter-responsive polymers offer a promising potential for targeting the age-related pathologies. Gasotransmitters are endogenously generated signalling molecules that readily diffuse across the cellular membranes for regulating diverse physiological processes and any alterations in their production or bioavailability potentially disrupts the cellular signalling networks and tissue homeostasis, thereby accelerating the ageing process. Hence, a dysregulated production of gasotransmitters during ageing may serve as an endogenous stimulus for the development of responsive polymeric systems, which selectively respond to pathological changes within ageing tissues. Among the gasotransmitters, the role of NO is well-established in ageing-associated endothelial dysfunction, inflammaging, neurodegeneration, and metabolic decline. Consequently, efforts have been focused on engineering NO-responsive polymeric systems for endogenous sensing of age-related alterations in NO bioavailability.

Several studies suggest a substantial age-associated decline in endothelial NO bioavailability, although the magnitude varies depending on tissue type, sex, and underlying health status. Both NO deficiency and excessive NO production can contribute to pathology, suggesting that future NO-responsive systems should be designed either to sense, scavenge, or restore NO depending on the specific ageing microenvironment. The age-associated NO decline is primarily attributed to the dysfunction of L-arginine-NO pathway which is caused by the uncoupling of nitric oxide synthase (NOS) resulting in hyper production of superoxide (instead of NO), along with the upregulation of arginase which further reduces NO bioavailability. These events drive chronic geriatric diseases, including vascular ageing (hypertension), atherosclerosis, and thrombosis (antiplatelet effect of NO). Growing evidence suggests an association between low NO levels and the Alzheimer's disease, where the accumulation of amyloid-beta (Aβ) peptides inhibits NO signalling, thereby accelerating disease progression. This is further substantiated by anomalous S-nitrosylation of proteins such as Cdk5 which contributes to synaptic loss and neurotoxicity (Pourbagher-Shahri et al., 2021[6]).

Despite its biological significance, the endogenous levels of NO cannot be directly measured by the routine clinical testing owing to its transient nature and lack of standardised assays. Flow-mediated dilatation has been the only accepted method so far for the clinical measurement of endothelial NO production, while the ongoing investigations also suggest salivary and urinary NOx as the potential biomarkers. The contemporary research on NO detection has evolved from the indirect organic methods to the development of metal-based systems designed for live cell imaging of NO. The direct sensing of NO has been achieved by fluorophore displacement (complexes of Co(II), Fe(II), Ru(II), and Rh(II)), metal reduction (observed in Cu(II) probes and conjugated polymer films), and reductive nitrosylation with CuFL1 (fluorescein complex with Cu(II)) which provides a rapid, selective and direct signalling of endogenous NO in various cell lines (Mazuryk et al., 2024[5]).

In the presence of dioxygen, NO has been reported to show reactivity towards O-phenylenediamine (O-phen) group to produce benzotriazole moiety, thereby triggering an irreversible fluorescence response. This chemical transformation has led to the development of NO-sensitive probes, metal complexes, fluorescence sensing molecules, and polymeric materials. NO-responsive monomers N-(2-aminophenyl) methacrylamide hydrochloride (NAPMA) and 2-(3-(2-aminophenyl) ureido) ethyl methacrylate hydrochloride (APUEMA) was developed by Hu et al. (2014[1]) which incorporated NO-sensing O-phen moiety. Eventually, NAPMA and APUEMA labelled biomimetic thermo-responsive copolymers were synthesised as P(NIPAM-co-NAPMA) and P(NIPAM-co-APUEMA) via reversible addition-fragmentation chain transfer (RAFT) polymerisation.

NO exposure induced pronounced optical and thermoresponsive changes in NAPMA- and APUEMA-based PNIPAM copolymers through benzotriazole formation. These polymers exhibited a detection limit of approximately 2.5 μM towards endogenous NO suggesting high sensitivity toward physiologically relevant NO concentrations. Modulation of the lower critical solution temperature (LCST) and triggered polymer self-assembly into hydrophobic nanostructures through chain collapse and aggregation were also observed in these polymers on NO stimulation. Such behaviour is particularly relevant to ageing, where alterations in NO homeostasis are closely associated with chronic inflammation, endothelial dysfunction, oxidative stress, and cellular senescence. By converting fluctuations in endogenous NO levels into measurable spectral, fluorescence, and thermoresponsive outputs, these copolymers provide a means to monitor age-associated microenvironmental changes. Furthermore, the fluorescence “turn-on” response, which is enhanced at physiological temperature, enables sensitive visualisation of NO-enriched senescent tissues, while NO-triggered self-assembly offers opportunities for site-specific drug delivery. Collectively, these polymers represent promising NO-responsive platforms for the diagnosis and treatment of ageing-related pathologies characterised by altered nitric oxide signalling (Thomas et al., 2008[9]).

Based on the same principle, Liu et al. (2020[3]) developed amphiphilic polypeptide comprising of poly(l-glutamate) fragment (PEOPA) functionalised with O-phen units on the glutamate side chains, serving as NO-trigger to manifest physicochemical changes in the parent polymeric chain.

The reported block polypeptide self-assembled into rigid one-dimensional (1D) filamentous nanostructures through the cooperative contribution of α-helical PEOPA segments and interchain hydrogen bonding among O-phen moieties. Unlike conventional amphiphilic block polypeptides that typically form spherical aggregates in aqueous media, this system exhibited highly ordered filamentous assemblies. NO exposure caused cyclisation of the O-phen pendants to benzotriazole intermediates, followed by their hydrolytic cleavage to generate a double-hydrophilic PEO-b-poly (L-glutamate) (PEO-b-PGlu) copolymer. This chemical transformation induced a progressive transition from an α-helical to a random coil conformation, disrupting the amphiphilic balance and weakening the interchain hydrogen-bonding network.

NO-induced structural rearrangement triggered rapid disassembly of the filamentous nanostructures. Exposure to an equimolar concentration of NO resulted in fragmentation of the long nanofilaments within 60 min, generating rod-like segments (40-280 nm) after 120 min and ultimately degrading them into fragments smaller than 20 nm after 180 min. This progressive disassembly was attributed to the loss of amphiphilicity, collapse of the helical architecture, and disruption of intermolecular interactions that collectively maintained the filamentous assemblies. Such NO-responsive behaviour is relevant to ageing-associated disorders, in which dysregulated NO signalling contributes to chronic inflammation, vascular dysfunction, and cellular senescence. The selective response of the polypeptides toward NO, with negligible interference from reactive oxygen, nitrogen, and sulfur species, indicates their potential for controlled gasotransmitter-responsive drug delivery. This high selectivity may reduce off-target activation in complex pathological microenvironments where multiple reactive species coexist, enabling more precise cargo release under conditions of altered NO signalling. Together with their structural stability and biocompatibility, these characteristics support the potential application of this nanoplatform for targeted therapeutic delivery in ageing-related diseases.

NO-responsiveness of O-phen moiety was further utilised by Latha et al. (2020[2]) for the development of polymeric, NO-responsive nano sensors aimed at fluorescence imaging of NO in living cells.

The reported NO-responsive nanoprobe exhibited favourable physicochemical characteristics, including a hydrodynamic diameter of approximately 170 nm and a near-neutral surface charge, properties that support efficient cellular uptake and intracellular imaging applications. Importantly, the probe remained largely inactive under extracellular conditions but demonstrated a marked fluorescence enhancement in the presence of intracellular concentrations of glutathione (GSH), indicating selective activation within the reducing intracellular environment. This redox-dependent activation minimises background interference and enhances the specificity of NO detection in living cells.

The authors further demonstrated the biological applicability of the nano sensor using hydrogen peroxide-stimulated fibroblast-like synoviocytes (FLS), cells known to contribute to chronic inflammatory processes in osteoarthritis and rheumatoid arthritis. A concentration-dependent increase in fluorescence intensity was observed following probe treatment, confirming its ability to sensitively detect biologically generated intracellular NO. Such sensitivity is particularly relevant given the growing recognition of NO dysregulation as a hallmark of inflammatory and age-associated pathologies.

From an ageing perspective, the GSH-responsive activation strategy is especially noteworthy. Ageing is characterised by disturbances in cellular redox homeostasis, including alterations in GSH levels, increased oxidative stress, and aberrant NO signalling. By remaining inactive in extracellular environments and selectively activating within reductive intracellular compartments, the nanoprobe offers a valuable tool for investigating age-related changes in NO biology with improved spatial and biochemical specificity.

Beyond biosensing applications, NO-responsive polymeric nanomaterials are increasingly being explored for targeted therapeutic delivery. For example, NO-triggered hyaluronic acid-levofloxacin nanomicelles have been developed by Lu et al. (2020[4]) to exploit elevated NO levels at infection sites, enabling site-specific antibiotic release. Such systems highlight the broader potential of NO-responsive polymeric platforms as both diagnostic and therapeutic tools, with promising implications for the management of inflammatory, infectious, and age-associated diseases.

The reported HA-NO-LF nanomicelles showed a uniform nanoscale size, with an average hydrodynamic diameter of approximately 129 nm in DLS analysis, which is highly suitable for an efficient cellular uptake and passive accumulation at the inflamed or infected sites. Treatment of the reported HA-NO-LF nanomicelles with NO led to the release of levofloxacin, which was most rapid after 6 h of exposure, reaching 70 % cumulative release after 15 h. These nanomicelles showed a minimal leakage of levofloxacin in the absence of NO, thereby confirming a sustained, NO-triggered release phenomenon. *In vitro* investigation gram-positive *Staph aureus* suggested negligible inhibition of bacterial growth in the absence of NO, which dropped sharply on NO exposure as indicated by a significant reduction in the bacterial colonies, matching the effect of levofloxacin.

After confirming the NO-responsiveness of the nanomicelles, their cellular uptake and receptor involvement in macrophages were also examined with DiO-loaded nanomicelles which produced strong fluorescence in RAW264.7 cells after 3 h, thereby indicating efficient cellular internalisation. However, during the pretreatment of cells with free hyaluronic acid (HA), the fluorescence intensity decreased, showing that free HA competes for CD44 receptors and blocks receptor-mediated uptake. These findings supported that the reported HA-NO-LF nanomicelles enter macrophages primarily through CD44-dependent endocytosis. To further assess NO-responsive behaviour under inflammatory conditions, RAW 264.7 macrophages, which naturally produce NO when infected were used as an *in vitro* model. Free levofloxacin effectively reduced intracellular bacteria, while a simple HA-LF mixture showed no added benefit, confirming that HA alone provides no antibacterial effect. In contrast, the reported HA-NO-LF nanomicelles produced a significant reduction in bacterial colony forming unit (CFU), owing to their uptake via HA-receptor-mediated endocytosis, followed by the release of LF from these nanomicelles inside the NO-rich macrophages. On blocking the NO production using L-NAME, a considerable dropping of the antibacterial activity of the nanomicelles was observed, which confirms that their therapeutic action depends on NO-triggered drug release. Finally, the *in vivo* antibacterial efficacy of the reported nanomicelles was conducted on *S. aureus*-infected mice which were treated intravenously with different formulations. HA-NO-LF nanomicelles produced the greatest reduction in bacterial burden, outperforming free LF and the HA+LF mixture, which showed no added benefit over LF alone. Histological analysis further demonstrated that HA-NO-LF markedly reduced lung inflammation and tissue damage compared to other groups. Moreover, an enhanced accumulation of the reported nanomicelles was observed at the infection site owing to a sharp fluorescence in the lung tissues. Hence, these observations showed that HA-NO-LF nanomicelles effectively target the infected tissues with promising therapeutic outcomes.

From an ageing and NO-signalling perspective, the HA-NO-LF nanomicelles are particularly relevant because they exploit NO-rich inflammatory microenvironments that are strongly linked to ageing-associated immune dysfunction (“inflammaging”). Their CD44-mediated macrophage targeting enables selective delivery into activated or senescent immune cells, where NO levels are dysregulated. Importantly, NO-triggered drug release ensures on-demand therapeutic activation specifically in these oxidative, ageing-like inflammatory niches, making the system useful for probing and potentially modulating NO-driven pathological processes associated with ageing.

Recently, Rong et al. (2025[7]) developed NO-scavenging micelles for the elimination of excess NO produced in the inflamed airways of ovalbumin-induced allergic asthma models. The presence of O-Phen units within the hydrophobic block of the polymeric chain led to its self-assembly in aqueous media to form PNOSM with an average hydrodynamic diameter of 78 nm, which was capable of converting excess NO to a benzotriazole framework, thereby acting as NO-scavenging material. A LPS-triggered macrophage activation model was adopted for examining the behaviour of developed PNOSMs under inflammatory stress. The exposure of resting Mo macrophages to LPS led to their differentiation to the pro-inflammatory M1 phenotype. LPS stimulation was also observed to cause a significant enhancement in the production of NO as compared to the PBS control which indicates a successful induction of the inflammatory state. In contrast to the strong NO surge seen with LPS alone, macrophages treated with NOSMs did not show a significant enhancement in NO, indicating that NOSMs effectively scavenge excess nitric oxide. As expected, the NOS inhibitor L-NAME also reduced NO production, showing a statistically significant decrease relative to the PBS control. Together, these findings suggest that NOSMs have a strong NO-scavenging effect. Elevated NO is well known to contribute to asthma pathology and airway remodelling through several downstream signalling axes, including the NO-cGMP-PKG-TRPV1, NO-cGMP-PKG-MAPK, and NO-cAMP-PKA-CREB pathways. Earlier studies have also shown that circulating IL-6 levels are consistently higher in patients with asthma than in healthy populations, reinforcing IL-6 as a useful supportive biomarker. The NOSMs developed in this study lowered IL-6 production in macrophages, underscoring their potential utility in dampening inflammatory signalling relevant to asthma. Additionally, the LPS stimulation triggered a sharp rise in intracellular IL-6, whereas NOSM treatment significantly suppressed this increase, likely through the reduction of NO.

The levels of other cytokines, such as IFN-γ in the LPS and L-NAME groups, did not differ markedly from the PBS group, the NOSM-treated macrophages exhibited an increase in IFN-γ. This indicates that NOSMs not only limit excessive NO but also enhance macrophage capacity to produce IFN-γ, which is a key mediator in immune regulation. The high NO concentrations were also found to suppress IFN-γ secretion primarily produced by Th1 cells, thereby highlighting NO as a negative regulator of Th1 activity. Complementary *in vivo* experiments in mice showed that NOSMs lowered NO levels in plasma and lung tissue, reduced IL-13 production, and simultaneously promoted IFN-γ production.

To evaluate these findings in an allergic asthma model, mice were divided into PBS, OVA, and NOSM groups (0.1 mg mL^-1^). Following OVA sensitisation and airway challenge, the normal and OVA groups received PBS nebulisation, while the NOSM group received NOSMs once daily from day 35 to 39. On day 40, blood and tissues were collected. HE staining experiments revealed a pronounced inflammatory infiltration in the OVA group, including neutrophils, mast cells, and eosinophils accompanied by thickened and distorted bronchial mucosa and marked airway narrowing. These pathological changes were much milder in the NOSM-treated mice. PAS staining showed heavy goblet-cell hyperplasia and mucus overproduction in the OVA group, whereas the NOSM group displayed noticeably reduced goblet-cell numbers and mucus accumulation. Overall, NOSM treatment relieved airway inflammation, reduced mucus remodelling, and improved airway structure, largely through effective NO clearance.

Given the emerging importance of SIRT6 in asthma, an epigenetic regulator linked to stress signalling, inflammation, and airway remodelling, its expression in lung tissue was also investigated. Previous studies have shown that lowering SIRT6 can ameliorate OVA-induced asthma features. Consistent with this, the western blot results in this study revealed significantly elevated SIRT6 in the OVA group compared with both the normal and NOSM groups. The quantification of inflammatory markers in serum and lung tissue. IL-6, IL-13, and nitrite were all found to be markedly higher in OVA-challenged mice, whereas NOSM-treated mice showed levels comparable to the normal group. In contrast, IFN-γ was found to be significantly increased in the NOSM group relative to both OVA and normal controls. These data collectively demonstrate that NOSMs reduce SIRT6 expression, curb airway inflammation, suppress cytokine overproduction, limit mucus hypersecretion, and restore immune balance by lowering NO. Importantly, no abnormal weight changes or organ toxicity were observed during treatment, confirming the excellent safety and biocompatibility of the reported NOSMs *in vivo*.

Considering the role of NO dysregulation in vascular ageing, inflammaging, neurodegeneration, and metabolic decline, NO-responsive polymers provide a unique platform to exploit age-associated molecular changes for designing precision therapeutics. Such systems advance over the conventional stimuli-responsive materials by leveraging a biologically relevant signalling molecule, NO, which is directly linked to several hallmarks of ageing. NO-responsiveness of these systems enables selective activation within specific pathological microenvironments while minimising the unintended responses.

Nevertheless, the challenges in clinical translation of these intelligent materials still persist. These include heterogeneity of NO levels across tissues, and limited data from preclinical ageing models to establish clinically relevant NO thresholds for polymer activation. Hence, the future efforts may be focused on integrating sensing and therapeutic functions within unified platforms and exploring their applicability across a broader spectrum of ageing-associated disorders.

## Declaration

### Conflict of interest

The authors declare no conflict of interest.

### Artificial Intelligence (AI) - assisted technology

The authors declare that they have not used artificial intelligence for the writing of the manuscript.

## References

[1] Hu J, Whittaker MR, Duong H, Li Y, Boyer C, Davis TP (2014). Biomimetic polymers responsive to a biological signaling molecule: nitric oxide triggered reversible self-assembly of single macromolecular chains into nanoparticles. Angew Chem Int Ed Engl.

[2] Latha AV, Ayyappan M, Kallar AR, Kakkadavath RV, Victor SP, Selvam S (2020). Fluorescence imaging of nitric oxide in living cells using o-phenylenediamine-rhodamine based polymeric nanosensors. Mater Sci Eng C Mater Biol Appl.

[3] Liu R, Xu M, Yan Q (2020). Nitric Oxide-Biosignal-Responsive Polypeptide Nanofilaments. ACS Macro Lett.

[4] Lu C, Xiao Y, Liu Y, Sun F, Qiu Y, Mu H (2020). Hyaluronic acid-based levofloxacin nanomicelles for nitric oxide-triggered drug delivery to treat bacterial infections. Carbohydr Polym.

[5] Mazuryk O, Gurgul I, Oszajca M, Polaczek J, Kieca K, Bieszczad-Żak E (2024). Nitric Oxide Signaling and Sensing in Age-Related Diseases. Antioxidants (Basel).

[6] Pourbagher-Shahri AM, Farkhondeh T, Talebi M, Kopustinskiene DM, Samarghandian S, Bernatoniene J (2021). An Overview of NO Signaling Pathways in Aging. Molecules.

[7] Rong D, Fang S, Huo S, Lin P, Cheng J, Hong J (2025). Nitric oxide-scavenging micelles alleviate airway inflammation in asthma via Th2 cytokine modulation. Mater Adv.

[8] Shymborska Y, Budkowski A, Raczkowska J, Donchak V, Melnyk Y, Vasiichuk V (2024). Switching it Up: The Promise of Stimuli-Responsive Polymer Systems in Biomedical Science. Chem Rec.

[9] Thomas DD, Ridnour LA, Isenberg JS, Flores-Santana W, Switzer CH, Donzelli S (2008). The chemical biology of nitric oxide: implications in cellular signalling. Free Radic Biol Med.

